# Microwave-Assisted Improved Synthesis of Pyrrolo[2,3,4-*kl*]acridine and Dihydropyrrolo[2,3,4-*kl*]acridine Derivatives Catalyzed by Silica Sulfuric Acid

**DOI:** 10.3390/molecules18021613

**Published:** 2013-01-28

**Authors:** Chengpao Cao, Changliang Xu, Wei Lin, Xuemei Li, Minghua Hu, Juxian Wang, Zhibin Huang, Daqing Shi, Yucheng Wang

**Affiliations:** 1 Key Laboratory of Organic Synthesis of Jiangsu Province, College of Chemistry, Chemical Engineering and Materials Science, Soochow University, Suzhou 215123, China; 2 Institute of Medicinal Biotechnology, Chinese Academy of Medical Sciences and Peking Union, Medical College, Beijing 100050, China; 3 Center for Drug Evaluation, State Food and Drug Administration Office of Generic Drug Pharmaceutical Science, Beijing 100038, China

**Keywords:** pyrrolo[2,3,4-*kl*]acridine, silica sulfuric acid, microwave irradiation

## Abstract

An improved synthesis of multifunctionalized pyrrolo[2,3,4-*kl*]acridine derivatives with different substituted patterns using silica sulfuric acid (SSA) as a heterogeneous catalyst under microwave irradiation conditions was developed. The reaction could be conducted by using readily available and inexpensive substrates within short periods of 12–15 min. under microwave irradiation. Compared with the conventional methods, the remarkable advantages of this method are milder reaction conditions, operational simplicity, higher yields, short reaction times, and an environmentally friendly procedure.

## 1. Introduction

Acridine derivatives were primarily used as stains for dye manufacturing (e.g., acridine orange) until their fluorescence and chemiluminescence properties found numerous other applications [1,2,3]. Such acridines have demonstrated important biological activity [4], including activity against cancer [5] due to their ability to intercalate into DNA and disrupt unwanted cellular processes [6]. This unique property of acridines has been exploited in many areas of medicine. As a result, significant biological activity toward viruses [7], bacteria [8], parasites [9,10], fungus [11], Alzheimer’s disease [12], and HIV/AIDS [13] has also been reported. Pyrrolo[2,3,4-*kl*]acridine derivatives have been isolated from a *Plakortis* sponge and showed biological activities [14,15]. Although there have been some reports on the synthesis of these molecules [16,17], those methods require multistep syntheses. Recently, we [18] and Tu [19] reported the one-pot synthesis of pyrrolo[2,3,4-*kl*]acridine derivatives catalyzed by L-proline or AcOH, respectively. However, these methods required the use of toluene or acetic acid as solvents. Thus, there is a need for the development of concise and green methods for the construction of this heterocyclic skeleton.

The need to reduce the amount of toxic waste and by-product arising from chemical process requires increasing emphasis on the use of less toxic and environmentally compatible materials in the design of new synthetic methods. One of the most promising approaches in organic synthesis is the use of reusable heterogeneous catalysts because of their environmental, economical, and industrial aspects [20]. The development of efficient methods using recoverable and reusable catalysts is an important goal in organic synthesis. Up to now, several reusable and heterogeneous catalysts have been designed and used. One useful example is silica sulfuric acid (SSA), which has been widely studied in recent years [21,22,23], in a variety of reactions such as cross-Aldol condensation [24], deacylation [25], selective oxidation [26], Michael addition [27] and functional group protection [28]. In our previous works, SSA was used as an efficient catalyst for the acetylation of aldehydes and sugars [29]. As a continuation of our interest in organic reactions catalyzed by solid acids [29,30], herein, we report the microwave-assisted green synthesis of pyrrolo[2,3,4-*kl*]acridine-1-one derivatives catalyzed by SSA in ethanol.

## 2. Results and Discussion

**Scheme 1:**
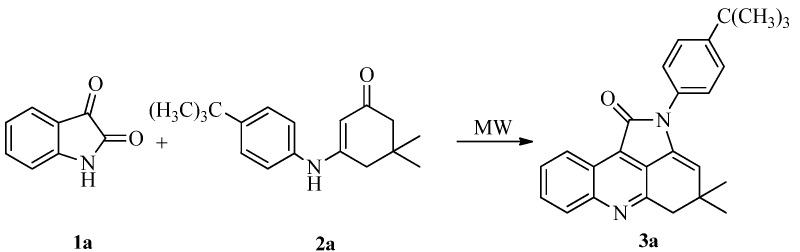
The model reaction.

In a preliminary study, we optimized the reaction conditions, including reaction solvents, temperature, catalyst and amount of SSA catalyst using isatin (**1a**), and 3-(4-*t*-butylphenylamino)-5,5-dimethylcyclohex-2-enone (**2a**) as model reactants (Scheme 1). The reaction mixture, which was composed of a 1:1 mixture of **1a** to **2a**, was tested under different conditions. The results are summarized in Table 1.

**Table 1:** Optimization of the reaction conditions for the synthesis of **3a**.

Entry	Solvent	Catalyst	T (°C)	Time (min)	Yield ^a^ (%)
1	ethanol	-	110	15	23
2	ethanol	SSA (0.02 g)	110	15	91
3	AcOH	SSA (0.02 g)	110	15	85
4	H_2_O	SSA (0.02 g)	110	15	81
5	ethylene glycol	SSA (0.02 g)	110	15	87
6	toluene	SSA (0.02 g)	110	15	91
7	ethanol	HCl (0.5 mL)	110	15	78
8	ethanol	H_2_SO_4_ (0.5 mL)	110	15	59
9	ethanol	I_2_ (0.02 g)	110	15	73
10	ethanol	L-proline (10 mol%)	110	15	40
11	ethanol	SSA (0.03 g)	110	15	90
12	ethanol	SSA (0.04 g)	110	15	91
13	ethanol	SSA (0.01 g)	110	15	76
14	ethanol	SSA (0.02 g)	90	15	71
15	ethanol	SSA (0.02 g)	100	15	85
16	ethanol	SSA (0.02 g)	120	15	87
17	ethanol	SSA (0.02 g)	110	8	77
18	ethanol	SSA (0.02 g)	110	10	87
19	ethanol	SSA (0.02 g)	110	12	91
20	ethanol	SSA (0.02 g)	110	20	89

^a^ Yield was determined by HPLC-MS.

The optimization process revealed that the reactions did not proceed in ethanol under catalyst-free conditions (Table 1, entry 1). Pleasingly, the target compound **3a** was obtained in ethanol with 0.02 g SSA as catalyst (Table 1, entry 2). To improve the yield, different solvents were evaluated. The results indicated that ethanol provided much better results than AcOH, ethylene glycol or water (Table 1, entries 2–5). The non-polar solvent toluene gave the same yield (Table 1, entry 6). Considering the toxicity of toluene, ethanol was selected as the preferred reaction solvent. Several other catalysts were also evaluated for their catalytic efficiency in the current reaction. Common acids (H_2_SO_4_ and HCl) and other catalysts (I_2_ or L-proline) can catalyze this reaction with low yields (Table 1, entries 7–10). The results revealed that SSA was the optimal catalyst with the product being isolated in 91% yield (Table 1, entry 2). Subsequently, we proceeded to evaluate the amount of SSA required for this reaction. When 0.02 g of silica gel was used, the reaction of **3a** proceeded in good yield (91%, Table 1, entry 2). The reaction yield remained unchanged when we increased the amount of SSA (Table 1, entries 11 and 12), but the yield was lower when the amount of SSA was decreased (Table 1, entry 13), therefore, 0.02 g of SSA is sufficient to initiate the reaction. To identify the optimum reaction temperature, the reaction was carried out with 0.02 g SSA at 90 °C, 100 °C, 110 °C and 120 °C, providing the product **3a** in yields of 71%, 85%, 91% and 87% (Table 1, entries 14, 15, 2 and 16), respectively, so the most suitable reaction temperature for this reaction is 110 °C. Finally, to optimize the reaction time, the reaction was carried out with 0.02 g SSA at 110 °C and the reaction time used was 8 min, 10 min, 12 min, 15 min and 20 min, respectively. It was found that the reaction can proceed smoothly in 12 min (Table 1, entry 19), while prolonging the reaction time did not enhance the yield of the product (Table 1, entries 2 and 20). Thus, the optimum conditions required the use of 0.02 g SSA as catalyst in ethanol as solvent at 110 °C and a reaction time of 12 min.

Having established the optimal conditions we proceeded to investigate the substrate scope of the transformation. As shown in Table 2, substituents such as bromo, chloro, fluoro on the isatin ring, and *t*-butyl or phenyl groups bearing either electron-withdrawing or electron-donating groups on the enaminone ring, were well tolerated under these reaction conditions, leading to the final products in satisfactory yields (up to 93%).

**Table 2:** Synthesis of dihydropyrrolo[2,3,4-*kl*]acridines **3**. 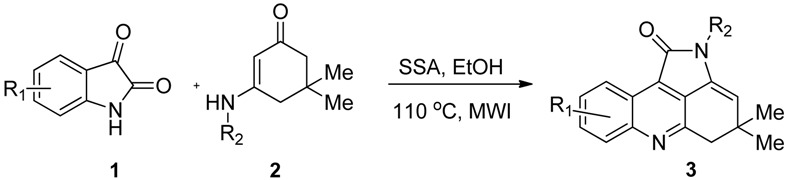

Entry	Product	R_1_	R_2_	Time (min)	Isolated Yield (%)
1	**3a**	H	4-*t*-BuC_6_H_4_	12	91
2	**3b**	H	3,5-(CH_3_)_2_C_6_H_3_	12	92
3	**3c**	H	2-CH_3_CH_2_C_6_H_4_	12	92
4	**3d**	H	3-Cl-4-FC_6_H_3_	15	89
5	**3e**	5-Cl	4-CH_3_OC_6_H_4_	12	90
6	**3f**	5-Cl	4-*t*-BuC_6_H_4_	12	91
7	**3g**	5-Cl	3,5-(CH_3_)_2_C_6_H_3_	12	92
8	**3h**	5-Cl	3-Cl-4-FC_6_H_3_	15	90
9	**3i**	5-Cl	2-CH_3_CH_2_C_6_H_4_	12	93
10	**3j**	5-Cl	*n*-C_4_H_9_	15	89
11	**3k**	5-F	4-CH_3_C_6_H_4_	12	92
12	**3l**	5-F	4-ClC_6_H_4_	12	91
13	**3m**	5-F	2,4-(CH_3_)_2_C_6_H_3_	12	91
14	**3n**	5-F	3-Cl-4-FC_6_H_3_	15	90
15	**3o**	5-F	4-*t*-BuC_6_H_4_	12	92
16	**3p**	5-F	2-CH_3_CH_2_C_6_H_4_	12	92
17	**3q**	5-Br	4-ClC_6_H_4_	12	93
18	**3r**	5-Br	3-Cl-4-FC_6_H_3_	15	90
19	**3s**	5-Br	4-BrC_6_H_4_	12	90
20	**3t**	5-Br	3,5-(CH_3_)_2_C_6_H_3_	12	91
21	**3u**	5-Br	*n*-C_4_H_9_	15	92

To expand the scope of the present method, *N*-substituted 3-aminocyclohex-2-enone, *N*-substituted 3-amino-5-phenylcyclohex-2-enones or *N*-substituted 3-amino-5-methylcyclohex-2-enones (**4**) were examined to replace the *N*-substituted 3-amino-5,5-dimethylcyclohex-2-enones (**2**), to our surprise, under the above optimized conditions, the desired 4,5-dihydropyrrolo[2,3,4-*kl*]acridine products **3** were not obtained. In the corresponding ^1^H-NMR spectra, the methylene group signals could not be detected, however a new aromatic proton could be detected easily. This result indicated that the corresponding oxidation products, the pyrrolo[2,3,4-*kl*]acridine derivatives **5** were produced (Table 3). The reason is that when there is no substituent or only one substituene (phenyl or methyl) on the C_5_ position of *N*-substituted 3-aminocyclohex-2-enone, the 4,5-dihydropyrrolo[2,3,4-*kl*]acridine derivatives would be oxidized by oxygen in the air to give pyrrolo[2,3,4-*kl*]acridine derivatives. The reaction pathways could therefore be controlled by varying the enaminones with different substitution patterns to give a series of novel 4,5-dihydropyrrolo[2,3,4-*kl*]acridin-1-ones **3** and pyrrolo[2,3,4-*kl*]acridin-1-ones **5** selectively.

**Table 3:** Synthesis of pyrrolo[2,3,4-*kl*]acridine derivatives **5**. 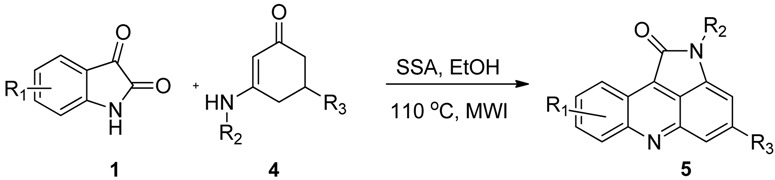

Entry	Product	R_1_	R_2_	R_3_	Time (min)	Isolated Yield (%)
1	**5a**	5-F	4-ClC_6_H_4_	H	15	87
2	**5b**	5-F	2,4-Cl_2_C_6_H_3_	H	15	88
3	**5c**	5-F	C_6_H_5_	H	15	87
4	**5d**	5-Cl	4-BrC_6_H_4_	H	15	88
5	**5e**	5-F	4-*t*-BuC_6_H_4_	H	15	86
6	**5f**	5-F	4-ClC_6_H_4_	Ph	15	87
7	**5g**	5-F	3-Cl-4-FC_6_H_3_	Ph	15	84
8	**5h**	H	C_6_H_5_	Ph	15	88
9	**5i**	5-F	2,4-(CH_3_)_2_C_6_H_3_	Ph	15	85
10	**5j**	H	4-BrC_6_H_4_	Ph	15	87
11	**5k**	5-Cl	4-*t*-BuC_6_H_4_	Ph	15	85
12	**5l**	5-F	C_6_H_5_	Ph	15	84
13	**5m**	5-Br	4-CH_3_C_6_H_4_	Ph	15	85
14	**5n**	5-Br	4-OCH_3_C_6_H_4_	CH_3_	15	85

**Figure 1:**
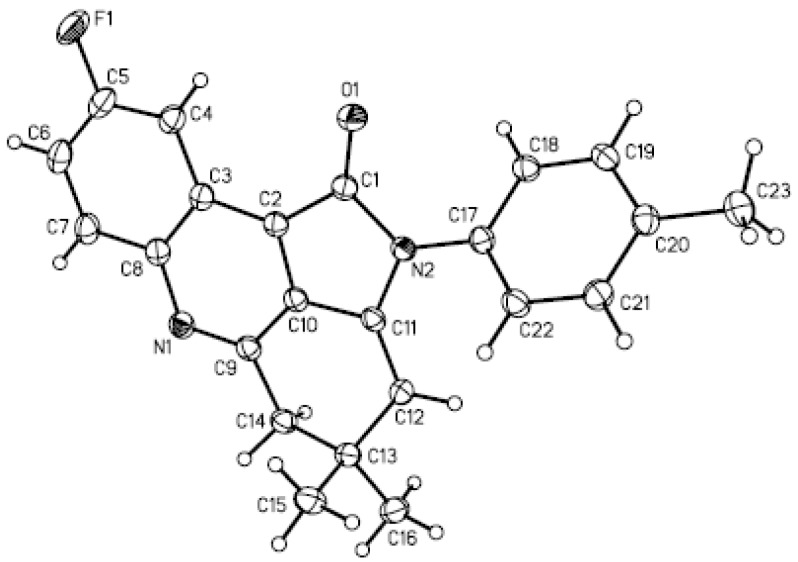
Molecular structure of compound **3k**.

The structures of the products **3** and **5**were identified from their IR, ^1^H-NMR, and HRMS spectra. The structure of compound **3k** was further confirmed by X-ray analysis (Figure 1).

## 3. Experimental

### 3.1. General

All reagents were purchased from commercial suppliers and used without further purification. Melting points are uncorrected. IR spectra were recorded on Varian F-1000 spectrometer in KBr with absorptions in cm^−1^. ^1^H-NMR (300 MHz or 400 MHz) spectra were recorded on a Varian Inova-300 MHz and Varian Inova-400 MHz (Palo Alto, CA, USA) in DMSO-*d_6_* or CDCl_3_ solution. *J* values are in Hertz. Chemical shifts are expressed in parts per million downfield from internal standard TMS. High-resolution mass spectra (HRMS) for all the compounds were determined on Bruker MicrOTOF-QII mass spectrometer with ESI resource (Billerica, MA, USA). X-ray crystallographic analysis was performed with a Rigaku Mercury CCD/AFC diffractometer (Tokyo, Japan). Microwave irradiation was carried out with an Initiator EXP Microwave Synthesizer from Biotage (Uppsala, Sweden). The reaction temperature was measured by an infrared detector during microwave heating.

### 3.2. General Procedure for the Synthesis of Compounds **3** and **5**

Isatin (**1**, 1.0 mmol) was introduced in a 10-mL Initiator reaction vial, and enaminone **2** or **4** (1.0 mmol) and 0.02 g of silica sulfuric acid as well as ethanol (3 mL) were then successively added. Subsequently, the reaction vial was closed and prestirred for 10 s. The mixture was irradiated (time, 12 or 15 min; temperature, 110 °C; absorption level, high; fixed hold time) until TLC (petroleum ether/ethyl acetate 3:1) revealed that convension of the starting material **1** was complete. The reaction mixture was then cooled to room temperature and concentrated. The solid was collected by Büchner filtration and purified by flash column chromatography (silica gel, mixture of petroleum ether/ethyl acetate, 3:1, v/v) to afford the desired products **3** or **5**.

*2-(4-tert-Butylphenyl)-4,5-dihydro-4,4-dimethylpyrrolo[2,3,4-kl]acridin-1(2H)-one* (**3a**). M.p. 168–170 °C; IR (KBr, cm^−1^) 2930, 1690, 1448, 1332, 1075, 959, 891, 831, 700; ^1^H-NMR (400 MHz, DMSO-*d_6_*): *δ* (ppm) 1.28 (s, 6H, 2 × CH_3_), 1.34 (s, 9H, (CH_3_)_3_), 3.33 (s, 2H, CH_2_), 5.80 (s, 1H, CH), 7.44 (d, *J* = 8.4 Hz, 2H, ArH), 7.59 (d, *J* = 8.4 Hz, 2H, ArH), 7.77 (t, *J* = 7.6 Hz, 1H, ArH), 7.84–7.88 (m, 1H, ArH), 8.16 (d, *J* = 8.8 Hz, 1H, ArH), 8.57 (d, *J* = 8.0 Hz, 1H, ArH). HRMS (ESI): *m/z* calcd. for C_26_H_27_N_2_O [M+H]^+^, 383.2123; found, 383.2103.

*4,5-Dihydro-4,4-dimethyl-2-(3,5-dimethylphenyl)pyrrolo[2,3,4-kl]acridin-1(2H)-one* (**3b**). M.p. 205–207 °C; IR (KBr, cm^−1^) 3064, 1717, 1634, 1499, 1386, 1327, 1257, 1186, 1092, 1018, 777; ^1^H-NMR (400 MHz, DMSO-*d_6_*): *δ* (ppm) 1.20 (s, 6H, 2 × CH_3_), 2.30 (s, 6H, 2 × CH_3_), 3.07 (s, 2H, CH_2_), 5.64 (s, 1H, CH), 7.02–7.04 (m, 3H, ArH), 7.64 (t, *J* = 7.2 Hz, 1H, ArH), 7.72 (t, *J* = 7.6 Hz, 1H, ArH), 8.04 (d, *J* = 8.0 Hz, 1H, ArH), 8.46 (d, *J* = 7.6 Hz, 1H, ArH). HRMS (ESI): *m/z* calcd. for C_24_H_23_N_2_O [M+H]^+^, 355.1810; found, 355.1811.

*2-(2-Ethylphenyl)-4,5-dihydro-4,4-dimethylpyrrolo[2,3,4-kl]acridin-1(2H)-one* (**3c**). M.p. 196–198 °C; IR (KBr, cm^−1^) 2957, 1708, 1646, 1500, 1348, 1146, 1083, 1016, 896, 830, 738; ^1^H-NMR (300 MHz, CDCl_3_): *δ* (ppm) 1.39–1.40 (m, 9H, 3 × CH_3_), 2.39–2.43 (m, 2H, CH_2_), 3.32 (s, 2H, CH_2_), 5.82 (s, 1H, CH), 7.33–7.39 (m, 2H, ArH), 7.59–7.60 (m, 1H, ArH), 7.73–7.82 (m, 3H, ArH), 8.27–8.30 (m, 1H, ArH), 8.72–8.74 (m, 1H, ArH). HRMS (ESI): *m/z* calcd. for C_24_H_23_N_2_O [M+H]^+^, 355.1810; found, 355.1823.

*2-(3-Chloro-4-fluorophenyl)-4,5-dihydro-4,4-dimethylpyrrolo[2,3,4-kl]acridin-1(2H)-one* (**3d**). M.p. 168–169 °C; IR (KBr, cm^−1^) 2958, 1710, 1462, 1356, 1171, 1100, 880, 755; ^1^H-NMR (300 MHz, CDCl_3_): *δ* (ppm) 1.34 (s, 6H, 2 × CH_3_), 3.26 (s, 2H, CH_2_), 5.60 (s, 1H, CH), 7.31–7.38 (m, 2H, ArH), 7.56 (s, 1H, ArH), 7.69–7.78 (m, 2H, ArH), 8.20–8.21 (m, 1H, ArH), 8.69–8.70 (m, 1H, ArH). HRMS (ESI): *m/z* calcd. for C_22_H_17_ClFN_2_O [M+H]^+^, 379.1013; found, 379.1015.

*9-Chloro-4,5-dihydro-2-(4-methoxyphenyl)-4,4-dimethylpyrrolo[2,3,4-kl]acridin-1(2H)-one* (**3e**). M.p. 162–164 °C; IR (KBr, cm^−1^) 2954, 1704, 1494, 1342, 1121, 1009, 819; ^1^H-NMR (300 MHz, CDCl_3_): *δ* (ppm) 1.32 (s, 6H, 2 × CH_3_), 3.18 (s, 3H, OCH_3_), 3.87 (s, 2H, CH_2_), 5.59 (s, 1H, CH), 7.04 (d, *J* = 6.6 Hz, 2H, ArH), 7.38 (d, *J* = 6.6 Hz, 2H, ArH), 7.65 (d, *J* = 7.2 Hz, 1H, ArH), 8.05 (d, *J* = 8.7 Hz, 1H, ArH), 8.66 (s, 1H, ArH). HRMS (ESI): *m/z* calcd. for C_23_H_20_ClN_2_O_2_ [M+H]^+^, 391.1213; found, 391.1194.

*2-(4-Tert-butylphenyl)-9-chloro-4,5-dihydro-4,4-dimethylpyrrolo[2,3,4-kl]acridin-1(2H)-one* (**3f**). M.p. 182–184 °C; IR (KBr, cm^−1^) 2947, 1575, 1485, 1392, 1319, 1253, 1077, 989, 833, 717; ^1^H-NMR (300 MHz, CDCl_3_): *δ* (ppm) 1.33 (s, 6H, 2 × CH_3_), 1.38 (s, 9H, (CH_3_)_3_), 3.19 (s, 2H, CH_2_), 5.68 (s, 1H, CH), 7.42 (s, 2H, ArH), 7.56–7.57 (m, 2H, ArH), 7.66–7.68 (m, 1H, ArH), 8.08–8.09 (m, 1H, ArH), 8.69 (s, 1H, ArH). HRMS (ESI): *m/z* calcd. for C_26_H_26_ClN_2_O [M+H]^+^, 417.1734; found, 417.1768.

*9-Chloro-4,5-dihydro-4,4-dimethyl-2-(3,5-dimethylphenyl)pyrrolo[2,3,4-kl]acridin-1(2H)-one* (**3g**). M.p. 178–180 °C; IR (KBr, cm^−1^) 2928, 1705, 1636, 1521, 1474, 1396, 1254, 1176, 1109, 1035, 803; ^1^H-NMR (400 MHz, DMSO-*d_6_*): *δ* (ppm) 1.11 (s, 6H, 2 × CH_3_), 2.21 (s, 6H, 2 × CH_3_), 2.97 (s, 2H, CH_2_), 5.61 (s, 1H, CH), 6.94 (s, 3H, ArH), 7.61–7.65 (m, 1H, ArH), 7.91 (d, *J* = 8.8 Hz, 1H, ArH), 8.23 (s, 1H, ArH). HRMS (ESI): *m/z* calcd. for C_24_H_22_ClN_2_O [M+H]^+^, 389.1421; found, 389.1405.

*9-Chloro-2-(3-chloro-4-fluorophenyl)-4,5-dihydro-4,4-dimethylpyrrolo[2,3,4-kl]acridin-1(2H)-one* (**3h**). M.p. 164–166 °C; IR (KBr, cm^−1^) 2944, 1634, 1484, 1369, 1288, 1182, 1093, 951, 818, 693; ^1^H-NMR (300 MHz, CDCl_3_): *δ* (ppm) 1.34 (s, 6H, 2 × CH_3_), 3.20 (s, 2H, CH_2_), 5.63 (s, 1H, CH), 7.32–7.37 (m, 2H, ArH), 7.57 (s, 1H, ArH), 7.69 (d, *J* = 7.8 Hz, 1H, ArH), 8.09 (d, *J* = 7.8 Hz, 1H, ArH), 8.66 (s, 1H, ArH). HRMS (ESI): *m/z* calcd. for C_22_H_16_ Cl_2_FN_2_O [M+H]^+^, 413.0624; found: 413.0608.

*9-Chloro-2-(2-ethylphenyl)-4,5-dihydro-4,4-dimethylpyrrolo[2,3,4-kl]acridin-1(2H)-one* (**3i**). M.p. 198–200 °C; IR (KBr, cm^−1^) 2948, 1703, 1638, 1516, 1457, 1351, 1209, 1087, 910, 879, 804; ^1^H-NMR (300 MHz, CDCl_3_): *δ* (ppm) 1.14–1.16 (m, 3H, CH_3_), 1.31 (s, 6H, 2 × CH_3_), 2.56 (s, 2H, CH_2_), 3.20 (s, 2H, CH_2_), 5.29 (s, 1H, CH), 7.27 (s, 1H, ArH), 7.36 (s, 1H, ArH), 7.45 (s, 2H, ArH), 7.67 (d, *J* = 8.1 Hz, 1H, ArH), 8.09 (d, *J* = 6.9 Hz, 1H, ArH), 8.69 (s, 1H, ArH). HRMS (ESI): *m/z* calcd. for C_24_H_22_ClN_2_O [M+H]^+^, 389.1421; found, 389.1416.

*2-Butyl-9-chloro-4,5-dihydro-4,4-dimethylpyrrolo[2,3,4-kl]acridin-1(2H)-one* (**3j**). M.p. 148–149 °C; IR (KBr, cm^−1^) 2950, 1706, 1500, 1346, 1240, 1091, 956, 890, 824, 697; ^1^H-NMR (300 MHz, CDCl_3_): *δ* (ppm) 0.92–0.94 (m, 3H, CH_3_), 1.29–1.36 (m, 8H, CH_2_ + 2 × CH_3_), 1.66–1.67 (m, 2H, CH_2_), 3.09–3.10 (m, 2H, CH_2_), 3.73–3.74 (m, 2H, CH_2_), 5.51 (s, 1H, CH), 7.57–7.58 (m, 1H, ArH), 7.96–7.98 (m, 1H, ArH), 8.56 (s, 1H, ArH). HRMS (ESI): *m/z* calcd. for C_20_H_22_ClN_2_O [M+H]^+^, 341.1421; found, 341.1412.

*2-(4-tert-Butylphenyl)-9-fluoro-4,5-dihydro-4,4-dimethylpyrrolo[2,3,4-kl]acridin-1(2H)-one* (**3k**). M.p. 168–170 °C; IR (KBr, cm^−1^) 3079, 1711, 1635, 1504, 1403, 1256, 1119, 1063, 775, 707; ^1^H-NMR (400 MHz, DMSO-*d_6_*): *δ* (ppm) 1.24 (s, 6H, 2 × CH_3_), 2.38 (s, 3H, CH_3_), 3.08 (s, 2H, CH_2_), 5.70 (s, 1H, CH), 7.31–7.36 (m, 4H, ArH), 7.60 (s, 1H, ArH), 8.00–8.09 (m, 2H, ArH). HRMS (ESI): *m/z* calcd. for C_23_H_20_FN_2_O [M+H]^+^, 359.1560; found, 359.1544.

*2-(4-Chlorophenyl)-9-fluoro-4,5-dihydro-4,4-dimethylpyrrolo[2,3,4-kl]acridin-1(2H)-one* (**3l**). M.p. 192–195 °C; IR (KBr, cm^−1^) 2957, 1703, 1508, 1360, 1223, 1110, 825, 699; ^1^H-NMR (300 MHz, CDCl_3_): *δ* (ppm) 1.32 (s, 6H, 2 × CH_3_), 3.19 (s, 2H, CH_2_), 5.63 (s, 1H, CH), 7.44–7.52 (s, 5H, ArH), 8.12–8.16 (m, 1H, ArH), 8.28–8.31 (m, 1H, ArH). HRMS (ESI): *m/z* calcd. for C_22_H_17_ClFN_2_O [M+H]^+^, 379.1013; found, 379.1005.

*9-Fluoro-4,5-dihydro-4,4-dimethyl-2-(2,4-dimethylphenyl)pyrrolo[2,3,4-kl]acridin-1(2H)-one* (**3m**). M.p. 148–150 °C; IR (KBr, cm^−1^) 2940, 1697, 1602, 1454, 1354, 1251, 1096, 970, 896, 834, 707; ^1^H-NMR (300 MHz, CDCl_3_): *δ* (ppm) 1.28 (s, 6H, 2 × CH_3_), 2.06 (s, 3H, CH_3_), 2.35 (s, 3H, CH_3_), 3.17 (s, 2H, CH_2_), 5.28 (s, 1H, CH), 7.13 (s, 1H, ArH), 7.24 (s, 2H, ArH), 7.46–7.48 (m, 1H, ArH), 8.12–8.14 (m, 1H, ArH), 8.28–8.31 (m, 1H, ArH). HRMS (ESI): *m/z* calcd. for C_24_H_22_FN_2_O [M+H]^+^, 373.1716; found, 373.1699.

*2-(3-Chloro-4-fluorophenyl)-9-fluoro-4,5-dihydro-4,4-dimethylpyrrolo[2,3,4-kl]acridin-1(2H)-one* (**3n**). M.p. 172–173 °C; IR (KBr, cm^−1^) 2958, 1613, 1473, 1363, 1273, 1171, 1070, 998, 910, 827, 701; ^1^H-NMR (300 MHz, CDCl_3_): *δ* (ppm) 1.33 (s, 6H, 2 × CH_3_), 3.22 (s, 2H, CH_2_), 5.63 (s, 1H, CH), 7.30–7.36 (m, 2H, ArH), 7.48–7.57 (m, 2H, ArH), 8.14–8.18 (m, 1H, ArH), 8.25–8.27 (m, 1H, ArH). HRMS (ESI): *m/z* calcd. for C_22_H_16_ClF_2_N_2_O [M+H]^+^, 397.0919; found, 397.0927.

*2-(4-tert-Butylphenyl)-9-fluoro-4,5-dihydro-4,4-dimethylpyrrolo[2,3,4-kl]acridin-1(2H)-one* (**3o**). M.p. 204–206 °C; IR (KBr, cm^−1^) 2950, 1707, 1639, 1493, 1348, 1201, 1086, 1017, 955, 833; ^1^H-NMR (400 MHz, DMSO-*d_6_*): *δ* (ppm) 1.19 (s, 6H, 2 × CH_3_), 1.27 (s, 9H, (CH_3_)_3_), 3.04 (s, 2H, CH_2_), 5.70 (s, 1H, CH), 7.36–7.37 (m, 2H, ArH), 7.51–7.59 (m, 3H, ArH), 7.97 (s, 1H, ArH), 8.07 (s, 1H, ArH). HRMS (ESI): *m/z* calcd. for C_26_H_26_FN_2_O [M+H]^+^, 401.2029; found, 401.2034.

*2-(2-Ethylphenyl)-9-fluoro-4,5-dihydro-4,4-dimethylpyrrolo[2,3,4-kl]acridin-1(2H)-one* (**3p**). M.p. 166–168 °C; IR (KBr, cm^−1^) 2949, 1699, 1514, 1353, 1196, 1098, 1017, 943, 843, 785; ^1^H-NMR (300 MHz, CDCl_3_): *δ* (ppm) 1.12–1.17 (m, 3H, CH_3_), 1.30 (s, 6H, 2 × CH_3_), 2.53–2.56 (m, 2H, CH_2_), 3.20 (s, 2H, CH_2_), 5.28 (s, 1H, CH), 7.27 (s, 1H, ArH), 7.35 (s, 1H, ArH), 7.44–7.46 (m, 2H, ArH), 7.50–7.53 (m, 1H, ArH), 8.15–8.18 (m, 1H, ArH), 8.33 (d, *J* = 6.3 Hz, 1H, ArH). HRMS (ESI): *m/z* calcd. for C_24_H_22_FN_2_O [M+H]^+^, 373.1716; found, 373.1712.

*9-Bromo-2-(4-chlorophenyl)-4,5-dihydro-4,4-dimethylpyrrolo[2,3,4-kl]acridin-1(2H)-one* (**3q**). M.p. 192–194 °C; IR (KBr, cm^−1^) 2949, 1705, 1499, 1351, 1102, 961, 826, 740; ^1^H-NMR (300 MHz, CDCl_3_): *δ* (ppm) 1.32 (s, 6H, 2 × CH_3_), 3.17 (s, 2H, CH_2_), 5.64 (s, 1H, CH), 7.44–7.49 (m, 4H, ArH), 7.80 (s, 1H, ArH), 7.97 (s, 1H, ArH), 8.81 (s, 1H, ArH). HRMS (ESI): *m/z* calcd. for C_22_H_17_BrClN_2_O [M+H]^+^, 439.0213; found, 439.0197.

*9-Bromo-2-(3-chloro-4-fluorophenyl)-4,5-dihydro-4,4-dimethylpyrrolo[2,3,4-kl]acridin-1(2H)-one* (**3r**). M.p. 168–170 °C; IR (KBr, cm^−1^) 2950, 1704, 1588, 1495, 1439, 1347, 1084, 822, 702; ^1^H-NMR (300 MHz, CDCl_3_): *δ* (ppm) 1.33 (s, 6H, 2 × CH_3_), 3.18 (s, 2H, CH_2_), 5.63 (s, 1H, CH), 7.30–7.36 (m, 2H, ArH), 7.56 (s, 1H, ArH), 7.79 (d, *J* = 6.6 Hz, 1H, ArH), 7.98 (d, *J* = 8.4 Hz, 1H, ArH), 8.77 (s, 1H, CH). HRMS (ESI): *m/z* calcd. for C_22_H_16_BrClFN_2_O [M+H]^+^, 457.0119; found, 457.0103.

*9-Bromo-2-(4-bromophenyl)-4,5-dihydro-4,4-dimethylpyrrolo[2,3,4-kl]acridin-1(2H)-one* (**3s**). M.p. 182–184 °C; IR (KBr, cm^−1^)2955, 1709, 1590, 1459, 1343, 1159, 1013, 889, 832, 766, 705; ^1^H-NMR (300 MHz, CDCl_3_): *δ* (ppm) 1.32 (s, 6H, 2 × CH_3_), 3.18 (s, 2H, CH_2_), 5.64 (s, 1H, CH), 7.37–7.39 (m, 2H, ArH), 7.65 (s, 2H, ArH), 7.79–7.82 (m, 1H, ArH), 7.99 (d, *J* = 6.9 Hz, 1H, ArH), 8.82 (s, 1H, CH). HRMS (ESI): *m/z* calcd. for C_22_H_17_ Br_2_N_2_O [M+H]^+^, 482.9708; found, 482.9693.

*9-Bromo-4,5-dihydro-4,4-dimethyl-2-(3,5-dimethylphenyl)pyrrolo[2,3,4-kl]acridin-1(2H)-one* (**3t**). M.p. 164–166 °C; IR (KBr, cm^−1^) 2958, 1915, 1612, 1550, 1445, 1318, 1229, 1101, 984, 823, 715; ^1^H-NMR (300 MHz, CDCl_3_): *δ* (ppm) 1.31 (s, 6H, 2 × CH_3_), 2.39 (s, 6H, 2 × CH_3_), 3.16 (s, 2H, CH_2_), 5.63 (s, 1H, CH), 7.03–7.07 (m, 3H, ArH), 7.22 (d, *J* = 6.0 Hz, 1H, ArH), 7.96–7.98 (m, 1H, ArH), 8.83 (s, 1H, CH). HRMS (ESI): *m/z* calcd. for C_24_H_22_BrN_2_O [M+H]^+^, 433.0916; found, 433.0906.

*9-Bromo-2-butyl-4,5-dihydro-4,4-dimethylpyrrolo[2,3,4-kl]acridin-1(2H)-one* (**3u**). M.p. 156–158 °C; IR (KBr, cm^−1^) 2938, 1699, 1510, 1450, 1356, 1245, 1126, 1028, 831, 789; ^1^H-NMR (300 MHz, CDCl_3_): *δ* (ppm) 0.95 (s, 3H, CH_3_), 1.32–138 (m, 8H, CH_2_ + 2 × CH_3_), 1.69–1.70 (m, 2H, CH_2_), 3.11–3.12 (m, 2H, CH_2_), 3.77 (s, 2H, CH_2_), 5.54 (s, 1H, CH), 7.74 (d, *J* = 7.2 Hz, 1H, ArH), 7.94 (d, *J* = 8.7 Hz, 1H, ArH), 8.78 (s, 1H, ArH). HRMS (ESI): *m/z* calcd. for C_20_H_22_BrN_2_O [M+H]^+^, 385.0916; found, 385.0917.

*2-(4-Chlorophenyl)-9-fluoropyrrolo[2,3,4-kl]acridin-1(2H)-one* (**5a**). M.p. 237–238 °C; IR (KBr, cm^−1^) 3022, 1716, 1638, 1185, 1074, 874; ^1^H-NMR (400 MHz, CDCl_3_): *δ* (ppm) 7.02–7.04 (m, 1H, ArH), 7.56–7.59 (m, 4H, ArH), 7.69–7.73 (m, 2H, ArH), 7.90 (d, *J* = 9.2 Hz 1H, ArH), 8.44–8.50 (m, 2H, ArH). HRMS (ESI): *m/z* calcd. for C_20_H_10_ClFN_2_NaO, 371.0363, [M+Na]^+^; found, 371.0345.

*2-(2,4-Dichlorophenyl)-9-fluoropyrrolo[2,3,4-kl]acridin-1(2H)-one* (**5b**). M.p. 248–250 °C; IR (KBr, cm^−1^) 3050, 1723, 1639, 1182, 1089, 785; ^1^H-NMR (400 MHz, CDCl_3_): *δ* (ppm) 6.66 (d, *J* = 6.8 Hz 1H, ArH), 7.43–7.45 (m, 1H, ArH), 7.58 (d, *J* = 8.0 Hz, 2H, ArH), 7.69–7.73 (m, 2H, ArH), 7.92 (d, *J* = 8.8 Hz 1H, ArH), 8.49–8.53 (m, 2H, ArH). HRMS (ESI): *m/z* calcd. for C_20_H_9_Cl_2_FN_2_NaO, 404.9974, [M+Na]^+^; found, 404.9989.

*9-Fluoro-2-phenylpyrrolo[2,3,4-kl]acridin-1(2H)-one* (**5c**). M.p. 228–230 °C; IR (KBr, cm^−1^) 2978, 1716, 1673, 1262, 1185, 1074, 874; ^1^H-NMR (400 MHz, CDCl_3_): *δ* (ppm) 7.03 (d, *J* = 6.8 Hz 1H, ArH), 7.46–7.47 (m, 1H, ArH), 7.60–7.76 (m, 6H, ArH), 7.87 (d, *J* = 8.8 Hz 1H, ArH), 8.43–8.50 (m, 2H, ArH). HRMS (ESI): *m/z* calcd. for C_20_H_11_FN_2_NaO, 337.0753, [M+Na]^+^; found, 337.0769.

*2-(4-Bromophenyl)-9-chloropyrrolo[2,3,4-kl]acridin-1(2H)-one* (**5d**). M.p. 218–220 °C; IR (KBr, cm^−1^) 2978, 1706, 1632, 1503, 1130, 1118 1096, 854; ^1^H-NMR (400 MHz, CDCl_3_): *δ* (ppm) 6.70–6.71 (m, 1H, ArH), 7.48–7.59 (m, 3H, ArH), 7.64–7.71 (m, 3H, ArH), 7.87–7.89 (m, 1H, ArH), 8.43–8.49 (m, 2H, ArH). HRMS (ESI): *m/z* calcd. for C_20_H_10_BrClN_2_NaO, 430.5963, [M+Na]^+^; found, 430.5978.

*2-(4-tert-Butylphenyl)-9-fluoropyrrolo[2,3,4-kl]acridin-1(2H)-one* (**5e**). M.p. 162–164 °C; IR (KBr, cm^−1^) 2951, 1710, 1628, 1512, 1353, 1205, 1096, 830, 718; ^1^H-NMR (300 MHz, CDCl_3_): *δ* (ppm) 1.42 (s, 9H, C(CH_3_)_3_), 7.04–7.06 (m, 1H, ArH), 7.57–7.71 (m, 6H, ArH), 7.85–7.89 (m, 1H, ArH), 8.44–8.51 (m, 2H, ArH). HRMS (ESI): *m/z* calcd. for C_24_H_20_FN_2_O, 370.1481, [M+H]^+^; found, 371.1537.

*2-(4-Chlorophenyl)-9-fluoro-4-phenylpyrrolo[2,3,4-kl]acridin-1(2H)-one* (**5f**). M.p. 238–240 °C; IR (KBr, cm^−1^) 2930, 2352, 1710, 1640, 1503, 1384, 1092, 805; ^1^H-NMR (300 MHz, CDCl_3_): *δ* (ppm) 7.46–7.69 (m, 11H, ArH), 8.04–8.05 (m, 1H, ArH), 8.41–8.42 (m, 2H, ArH). HRMS (ESI): *m/z* calcd. for C_26_H_15_ClFN_2_O, 425.0857, [M+H]^+^; found, 425.0846.

*2-(3-Chloro-4-fluorophenyl)-9-fluoro-4-phenylpyrrolo[2,3,4-kl]acridin-1(2H)-one* (**5g**). M.p. 230–232 °C; IR (KBr, cm^−1^) 3069, 2926, 2354, 1716, 1639, 1499, 1087, 812; ^1^H-NMR (300 MHz, CDCl_3_): *δ* (ppm) 7.29–7.54 (m, 6H, ArH), 7.70–7.76 (m, 4H, ArH), 8.01–8.02 (m, 1H, ArH), 8.42–8.45 (m, 2H, ArH). HRMS (ESI): *m/z* calcd. for C_26_H_14_ ClF_2_N_2_O, 443.0763 [M+H]^+^; found, 443.0770.

*2,4-Diphenylpyrrolo[2,3,4-kl]acridin-1(2H)-one* (**5h**). M.p. 220–221 °C; IR (KBr, cm^−1^) 2921, 1718, 1637, 1496, 1103, 1008, 761; ^1^H-NMR (400 MHz, CDCl_3_): *δ* (ppm) 7.27 (s, 1H, ArH), 7.45–7.53 (m, 4H, ArH), 7.60–7.73 (m, 6H, ArH), 7.80 (t, *J* = 8.0 Hz, 1H, ArH), 7.93 (t, *J* = 8.4 Hz, 1H, ArH), 8.06 (s, 1H, ArH), 8.42 (d, *J* = 8.8 Hz, 1H, ArH), 8.89 (d, *J* = 8.0 Hz, 1H, ArH). HRMS (ESI): *m/z* calcd. for C_26_H_16_N_2_NaO, 395.1160 [M+Na]^+^; found, 395.1182.

*9-Fluoro-2-(2,4-dimethylphenyl)-4-phenylpyrrolo[2,3,4-kl]acridin-1(2H)-one* (**5i**). M.p. 188–190 °C; IR (KBr, cm^−1^) 2990, 1705, 1633, 1131, 1066, 854; ^1^H-NMR (400 MHz, CDCl_3_): *δ* (ppm) 2.08 (s, 3H, CH_3_), 2.26 (s, 3H, CH_3_), 6.72–6.73 (m, 1H, ArH), 7.03–7.16 (m, 3H, ArH), 7.22–7.29 (m, 3H, ArH), 7.47–7.49 (m, 3H, ArH), 7.82–7.84 (m, 1H, ArH), 8.22–8.27 (m, 2H, ArH). HRMS (ESI): *m/z* calcd. for C_28_H_20_FN_2_O, 419.1560, [M+H]^+^; found, 419.1559.

*2-(4-Bromophenyl)-4-phenylpyrrolo[2,3,4-kl]acridin-1(2H)-one* (**5j**). M.p. 234–235 °C; IR (KBr, cm^−1^) *ν*2987, 1703, 1636, 1501, 1253, 1181, 1085, 832. ^1^H-NMR (CDCl_3_-*d_1_*, 400 MHz) *δ* : 7.22 (s, 1H, ArH), 7.40–7.56 (m, 5H, ArH), 7.69–7.79 (m, 5H, ArH), 7.91 (t, *J* = 7.6 Hz, 1H, ArH), 8.05 (s, 1H, ArH), 8.40 (d, *J* = 8.8 Hz, 1H, ArH), 8.83 (d, *J* = 8.4 Hz, 1H, ArH). HRMS (ESI): *m/z* calcd. for C_26_H_16_BrN_2_O, 451.0446, [M+H]^+^; found, 451.0409.

*2-(4-tert-Butylphenyl)-9-chloro-4-phenylpyrrolo[2,3,4-kl]acridin-1(2H)-one* (**5k**). M.p. 222–223 °C; IR (KBr, cm^−1^) 2922, 1719, 1634, 1383, 1171, 1077, 822; ^1^H-NMR (400 MHz, CDCl_3_): *δ* (ppm) 1.42 (s, 9H, (CH_3_)_3_), 7.19–7.22 (m, 1H, ArH), 7.42–7.68 (m, 9H, ArH), 7.75–7.80 (m, 1H, ArH), 7.92–7.96 (m, 1H, ArH), 8.27–8.31 (m, 1H, ArH), 8.73–8.78 (m, 1H, ArH) HRMS (ESI): *m/z* calcd. for C_30_H_24_ClN_2_O, 463.1577, [M+H]^+^; found, 463.1570.

*9-Fluoro-2,4-diphenylpyrrolo[2,3,4-kl]acridin-1(2H)-one* (**5l**). M.p. 218–219 °C; IR (KBr, cm^−1^) 2957, 1705, 1633, 1322, 1173, 1096, 854; ^1^H-NMR (400 MHz, CDCl_3_): *δ* (ppm) 7.43–7.51 (m, 4H, ArH), 7.59–7.70 (m, 8H, ArH), 8.01–8.02 (m, 1H, ArH), 8.41–8.43 (m, 2H, ArH). HRMS (ESI): *m/z* calcd. for C_26_H_16_FN_2_O, 391.1247, [M+H]^+^; found, 391.1229.

*9-Bromo-4-phenyl-2-p-tolylpyrrolo[2,3,4-kl]acridin-1(2H)-one* (**5m**). M.p. 220–222 °C; IR (KBr, cm^−1^) 2921, 1706, 1635, 1384, 1129, 1092, 820; ^1^H-NMR (400 MHz, CDCl_3_): *δ* (ppm) 2.45 (s, 3H, CH_3_), 7.17 (s, 1H, ArH), 7.38–7.50 (m, 7H, ArH), 7.65 (d, *J* = 7.6 Hz, 2H, ArH), 7.89–7.95 (m, 2H, ArH), 8.21 (d, *J* = 9.2 Hz, 1H, ArH), 8.98 (s, 1H, ArH). HRMS (ESI): *m/z* calcd. for C_27_H_17_BrN_2_NaO, 487.0422, [M+H]^+^; found, 487.0403.

*9-Bromo-2-(4-methoxyphenyl)-4-methylpyrrolo[2,3,4-kl]acridin-1(2H)-one* (**5n**). M.p. 216–218 °C; IR (KBr, cm^−1^) 2916, 1718, 1637, 1516, 1384, 1124, 1080, 849; ^1^H-NMR (400 MHz, CDCl_3_): *δ* (ppm) 2.50 (s, 3H, CH_3_), 2.60 (s, 3H, OCH_3_), 6.82 (s, 1H, ArH), 7.41–7.60 (m, 5H, ArH), 7.93–7.96 (m, 1H, ArH), 8.24–8.26 (m, 1H, ArH), 9.03–9.04 (m, 1H, ArH). HRMS (ESI): *m/z* calcd. for C_22_H_15_BrN_2_NaO_2_, 441.0215, [M+Na]^+^; found, 441.0218.

## 4. Conclusions

In conclusion, we have developed a procedure for the simple synthesis of a variety of potential biologically active pyrrolo[2,3,4-*kl*]acridines based on a novel domino reaction. Using this method, a library of molecularly diverse pyrrolo[2,3,4-*kl*]acridine derivatives was rapidly assembled (12–15 min) with excellent yields (84%–93%) by using readily available and inexpensive substrates under microwave irradiation.
